# Coherent collections of rules describing exceptional materials identified with a multi-objective optimization of subgroups†

**DOI:** 10.1039/d5dd00174a

**Published:** 2025-06-25

**Authors:** Lucas Foppa, Matthias Scheffler

**Affiliations:** a The NOMAD Laboratory at the Fritz Haber Institute of the Max Planck Society Faradayweg 4-6 D-14195 Berlin Germany foppa@fhi-berlin.mpg.de

## Abstract

Useful materials are often statistically exceptional and they might be overlooked by artificial intelligence (AI) models that attempt to describe all materials simultaneously. These global models perform well for the majority of materials, but they do not necessarily capture the useful ones. Subgroup discovery (SGD) identifies descriptions of subsets of materials associated with exceptional values of a chosen property. Thus, SGD can better capture exceptional materials compared to widely used AI techniques. Previous studies focused on the SG that maximizes an objective function establishing a tradeoff between the SG size and the exceptionality of the distribution of property values within the SG. However, this optimization does not give a unique solution, but many SGs typically have similar objective-function values. Here, we identify a “Pareto region” of SGD solutions presenting a multitude of size-exceptionality tradeoffs. The approach is demonstrated by learning descriptions of perovskites with a high bulk modulus.

## Abstract 2

Subgroup discovery identifies rules describing outstanding materials and highlighting key physical parameters correlated with materials' properties.

## Graphical abstract

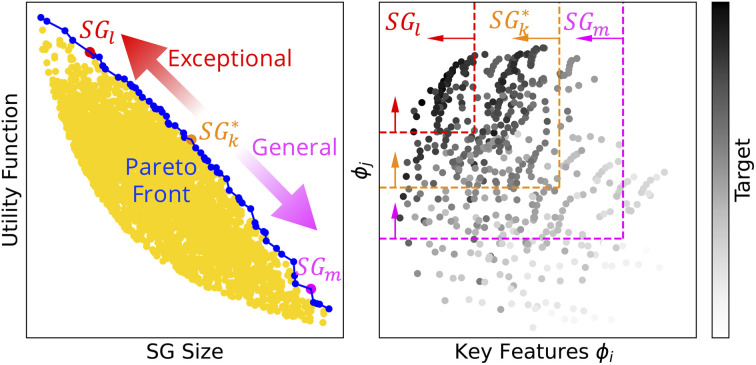

## 1 Introduction

Materials serve as the cornerstone of critical economic sectors and they play a pivotal role in driving the transition to a sustainable economy and to renewable energy.^1,2^ Thus, there is an urgent need for the discovery of appropriate and more efficient materials. However, the materials that are useful for a given application are often statistically exceptional. These materials might present, for instance, extremely high values of materials properties compared to other known compounds. Exceptional materials are very few compared to the practically infinite space of possible materials, which remains largely unknown.^3,4^ Artificial intelligence (AI) has been increasingly applied to identify correlations and patterns in data in materials science and engineering.^5–8^ Indeed, AI might describe materials properties and functions governed by intricate mechanisms better than previous theoretical and computational approaches because it targets correlations and does not assume a single underlying physical model.^9^ Thus, AI holds the potential to accelerate the exploration of the immense materials space, leading to the discovery of new materials. However, capturing exceptional materials is a challenging task for most widely used AI methods.

AI methods often fail in describing the exceptional materials first because the training data are typically not well distributed over (or representative of) the huge, unknown materials space. Therefore, interpolation schemes are unable to generalize to potentially interesting portions of the materials space that were disregarded in the training data.^10–14^ This issue, which might be referred to as an “out of distribution” issue, can be alleviated by AI approaches that can better extrapolate compared to methods that are inherently interpolative.^12,15^ Besides, AI model training can be combined with the systematic acquisition of new data corresponding to portions of the materials space that were not covered by the initial training data using sequential-learning approaches such as active learning or Bayesian optimization.^16–19^ However, the efficiency of sequential learning often relies on the quality of uncertainty estimates, which is in some cases problematic.^20–22^ A second key reason that can explain the inability of current AI approaches to capture exceptional materials is the focus on global models. These models attempt to describe all materials simultaneously. They are obtained by optimizing an objective (loss) function that reflects the average performance, *e.g.*, the mean prediction error. Thus, global models are designed to perform well in average for the majority of (uninteresting) materials, but do not necessarily perform well for exceptional ones.^23^ Objective functions can be adapted to give more importance to the description of specific property values, *e.g.*, high values.^24^ However, different groups of materials could operate according to different mechanisms. This might render a global description not only inaccurate, but also inappropriate.

Alternative AI methods for materials discovery include strategies based on similarity among materials^25,26^ or among their constituents, *e.g.*, ions in solids,^27^ and the subgroup-discovery (SGD)^28,29^ approach. In particular, SGD tackles the limitations of global descriptions and it has the potential to better capture exceptional materials. Indeed, SGD has been recently put forward in materials science.^30–35^ SGD is a supervised, descriptive rule-induction^36^ technique and it identifies subsets of a dataset associated with exceptional values of a target quantity of interest, for instance a materials property or performance indicator. Crucially, SGD identifies these subsets (SGs) of data along with the descriptions of these subsets, referred to as rules. The SGD analysis starts with the choice of many features that relate to possibly relevant mechanisms governing the target materials property. Then, SGD creates a number of statements about the features that are satisfied only for a part of the dataset. These statements are, for instance, inequalities constraining the values of the features. Finally, a search algorithm^37–39^ identifies the combination of typically few statements that results in a SG that maximizes an objective function. This objective (or quality) function is a product of the relative SG size and the so-called utility function. The relative SG size is the fraction of data points that satisfies the statements describing the SG. The higher the relative SG size, the more general the description. The utility function quantifies the “exceptionality” of the distribution of target values in the SG with respect to the entire dataset. The positive mean shift is one example of a utility function often utilized when the target values of interest are high. This utility function measures the shift of the mean value of the target in the SG with respect to the mean value of the target in the entire dataset. The higher the value of the positive-mean-shift utility function, the higher the target values in the identified SG. Thus, such a utility function favors the identification of SGs associated with high target values. We will discuss this utility function in more detail in the Results section. We will also discuss a second example of the utility function, namely the Jensen–Shannon divergence between the distributions of target values in the SG and in the entire dataset.

**Fig. 1:**
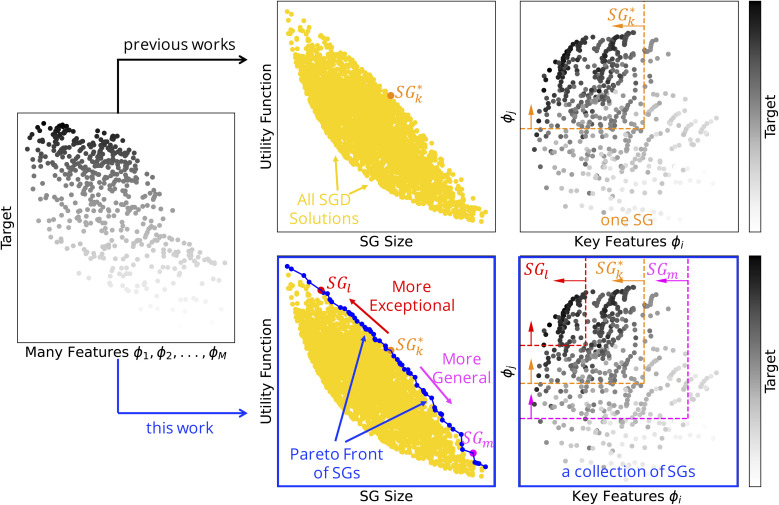
Subgroup discovery (SGD) identifies subsets of materials that are outstanding with respect to a certain materials property, the target of interest. The subsets, or subgroups (SGs), are described by rules that typically constrain key features characterizing the materials and mechanisms governing the target, out of many initially offered features. The subgroups are obtained by maximizing an objective function that is a product of the (relative) SG size and the utility function. These two terms reflect the generality and the exceptionality of the SG, respectively. Top: Previous studies focused on the one SG that maximizes the objective function, denoted as 
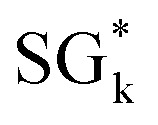
. Bottom: In this work, we identify a collection of SGs containing, *e.g.*, SG_l_, 
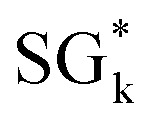
, and SG_m_, and presenting multiple tradeoffs between the SG size and the utility function.

SG rules typically constrain the values of only a few key features, out of the many initially offered ones. Thus, SGD learns a (low-dimensional) representation. The SGD approach is illustrated in Fig. 1 (top). The aim of SGD is to find descriptions of portions of the materials space that are exceptional. Thus, it accepts that the mechanisms governing the materials' properties might vary across the materials space and that not all of these mechanisms need to be described for the discovery of useful materials. Indeed, SGD was used to identify rules associated with high-performance materials even based on datasets dominated by low-performance situations.^35^ Additionally, SGD is an exploratory analysis that can identify unexpected patterns and anomalies. We note that SGD is significantly different from clustering techniques because it does not aim at describing the entire dataset. Moreover, SGD is a supervised approach, and it explicitly identifies rules indicating why the data points belong to the SG. Clustering is an unsupervised technique that groups data points into clusters based on similarity, without considering any target quantity. Besides, clustering does not explicitly identify why the data points are clustered together.

Previous SGD studies^30–33^ focused on the identification of the SG (and rules) that maximizes the objective function, as illustrated in Fig. 1 (top). However, the SG that maximizes the objective function does not reflect all possible tradeoffs between the relative SG size and utility function that could be relevant for a given application. Additionally, the definitions of some utility functions assume that the distributions of target values in the entire dataset and in the SG are appropriately characterized by one single summary-statistics value, such as the mean value in the case of the positive-mean-shift utility function. However, this assumption may be questioned in materials science, as the distributions can significantly deviate from the normal distribution. Indeed, distributions related to materials can be skewed or even bimodal. Finally, utility functions often assume that the summary-statistics values appropriately reflect the huge, unknown materials space, *e.g.*, the mean value in the dataset is a good approximation for the mean value in the entire materials space. This assumption does not hold if the datasets are created according to certain selection biases. Thus, the datasets can be highly unbalanced compared to the materials space.

In this manuscript, we introduce a multi-objective optimization of SGs that identifies coherent collections of SGs and rules in the “Pareto region” of optimal SGD solutions. These SGs present a multitude of tradeoffs between the relative SG size and the utility function, the two conflicting objectives in SGD. This concept is schematically shown in Fig. 1 (bottom). The multi-objective optimization of SGs is demonstrated for the identification of *AB*O_3_ perovskites with a high bulk modulus as an example of a target. We compare the SGs obtained with two different utility functions, the positive mean shift and the cumulative Jensen–Shannon divergence. The latter does not make assumptions on the shape of the distributions of target values. We also analyze the sensitivity of the results with respect to the offered set of features. Finally, we exploit the rules trained on a dataset of 504 single *AB*O_3_ perovskites to identify high-bulk-modulus perovskites out of a candidate space of 12 096 single *AB*O_3_ and double *A*_2_*BB′*O_6_ perovskites. Our results show that rules focusing on perovskites with a high bulk modulus do not necessarily correspond to the single SG which maximizes the objective function, but they can be systematically derived with the Pareto-region concept. These rules identify perovskites of the candidate space that present the bulk modulus up to 13% higher than the highest value of the training set.

## 2 Methods

### 2.1 Subgroup discovery

The SGD approach is based on a dataset of materials, which we denote as P̃. This dataset is part of the huge materials space, the full population, P. Each material of the full population is associated with a set of features, namely physical parameters that are potentially related to a target quantity of interest *y*, for instance, a materials property. The target of interest is only known for the materials in the dataset. SGD starts by systematically constructing statements about the features. Each statement is only verified for a part of the materials in the dataset. Thus, the statements select part of the dataset. The construction of these statements follows different approaches depending on the type of feature: categorical, ordinal, or metric. For categorical features, *i.e.*, when the feature values are a discrete set with no relevant order, all possible statements of the form *ϕ* = *c*_*i*_ are constructed, where *c*_*i*_ are the categories in the dataset. For ordinal features, *i.e.*, when the feature values contain a set of discrete and ordered values, all possible inequality constraints such as *ϕ* ≥ *z*_*i*_ and *ϕ* ≤ *z*_*i*_ are generated, where *z*_*i*_ represents the integer values in the dataset. For metric features, *i.e.*, when the feature values are from a continuous ordered scale, statements similar to those of the ordinal case are constructed, *i.e.*, *ϕ* ≥ *ν*_*i*_ and *ϕ* ≤ *ν*_*i*_. In this case, however, one cannot simply use all possible *ν*_*i*_ values, but instead has to find a small computationally feasible subset of *ν*_*i*_ values. This is accomplished with the aid of *k*-means clustering. First, the clustering algorithm is applied to identify *k* + 1 values representing the center of clusters corresponding to range of values for each of the features in the dataset. Then, the arithmetic means between the centers of two neighboring clusters are taken as possible *ν*_*i*_. Thus, the possible *ν*_*i*_ values are closer to each other when the concentration of data is higher. In this work, we use *k* = 10. Further details on the construction of statements and on the choice of *k* are discussed elsewhere.^30,40^ Then, SGD uses a search algorithm, for instance Monte Carlo-based^37,38^ or branch-and-bound,^39^ to identify conjunctions of statements constructed with the “AND” operator (∧), that result in SGs that maximize an objective (quality) function *Q* of the form1
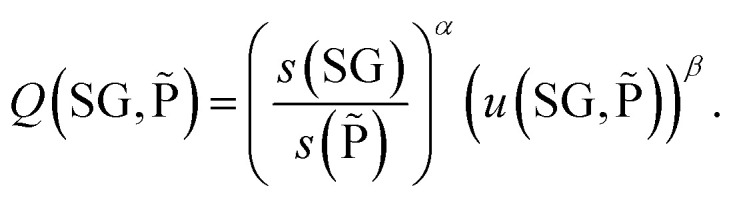
Here, *s*(SG) and *s*(P̃) are the sizes of the SG and of the dataset P̃, respectively, *i.e.*, the number of data points that satisfy the statements defining the SG and the number of data points in the entire dataset. The ratio between the size of the SG and the size of the dataset, *s*(SG)/*s*(P̃), is referred to as the relative SG size. *u*(SG, P̃) is the utility function describing how exceptional the distribution of the target in the SG is compared to the entire dataset. The utility function is chosen according to the question to be addressed, and there are many possibilities.^31^ The positive shift of the mean value of the target in the SG compared to the mean value of the target in the entire dataset and the Jensen–Shannon divergence between the distribution of target values in the SG and the distribution of target values in the entire dataset^41^ are two examples of utility functions that we will consider in this work. Finally, *α* and *β* are tunable parameters controlling the tradeoff between the relative SG size, *i.e.*, the generality of the description, and the utility function, *i.e.*, the exceptionality of the description. Usually, *α* = *β* = 1 or *β* = 1 − *α*, with *α* ∈ [0.1, 0.9]. The Monte Carlo search algorithm^37,38^ randomly generates conjunctions of the previously generated statements with probability proportional to 
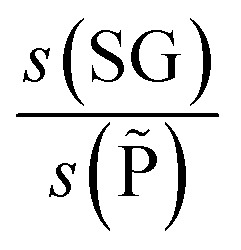
. Then, an opportunistic pruning algorithm refines these conjunctions by removing statements that result in the increase of *Q*(SG, P̃) values. The iterative removal of statements leads to the maximization of the objective function of eqn (1). We note that SG search algorithms such as the branch-and-bound approach^39^ are more systematic than the stochastic Monte Carlo algorithm. However, the computational cost of the branch-and-bound approach increases more rapidly with the number of statements compared to the cost of the Monte Carlo search. Finally, it should be noted that optimizing the SGD objective function over the full set of possible conjunctions of statements about the data is an NP-hard combinatorial optimization problem and that SG searches are extensive but not exhaustive. Thus, there is no guarantee that all possible SGs will be identified in the Pareto front of SGD solutions. In this work, we used the SGD algorithm implemented in the realkd version 0.7.2. A Monte Carlo-based SG search algorithm^37,38^ was used with 50 000 seeds for the initialization.

The inputs to the SGD analysis are the datasets containing a target quantity of interest (*e.g.*, a materials property) and the features that characterize the materials. Additionally, one has to choose an appropriate quality function, which determines the desired distribution of target values in the SGs. The outputs are the subsets of data (SGs) and the rules (statements) that describe these subsets of data. These rules typically depend only on key features, out of all initially offered features. In analogy to genes in biology, these key features might be called materials genes,^9^ as they correlate with the mechanisms governing the materials properties. The rules can be exploited to efficiently identify the few exceptional materials in the huge materials space P, for which the target property is unknown.

### 2.2 Approach for identifying the Pareto region of SGD solutions

In order to identify the pursued coherent collections of SGs with multiple generality-exceptionality tradeoffs, we first run the SGD algorithm using the objective function of eqn (1) with *α* = *β* = 1. Thus, relative SG size and the utility function are given the same importance. Then, we collect a number of SGD solutions identified by this algorithm that display high objective-function values. Among these top-ranked SGD solutions, we identify a Pareto front with respect to the two objectives relative SG size and the utility function. In multi-objective optimization, a Pareto front is the set of solutions for which no single objective can be improved without deteriorating at least one other objective. Thus, the solutions in the Pareto front reflect an optimal tradeoff between competing objectives. To ensure that no interesting SGD solution is left out, we included in our analysis not only solutions that are part of the Pareto front but also solutions within a fixed threshold distance (in this work equal to 0.01) to the Pareto front in the relative SG size-utility function space, *i.e.*, solutions which are near the Pareto front. We refer to the solutions at the Pareto front plus the solutions near the Pareto front as the *Pareto region*. The definition of a Pareto region *via* a fixed distance to the Pareto front ensures that all SGD solutions of the Pareto region have objective-function values within the range determined by the chosen threshold distance. However, this approach is sensitive to the form of the Pareto front and the distribution of SGD solutions in the Pareto front. This issue can be alleviated by defining the Pareto region based on subsequent Pareto fronts. This alternative approach to define the Pareto region is discussed in detail and compared with the distance-based approach in the ESI.†

## 3 Results and discussion

### 3.1 Identification of perovskites with a high bulk modulus

The identification of coherent collections of SGs and the usefulness of our approach will be demonstrated for the learning of rules describing the bulk modulus (*B*_0_) of *AB*O_3_ perovskites. More specifically, the problem that we will tackle is the identification of materials that exhibit a high bulk modulus. The bulk modulus quantifies the resistance of the material to compression and it correlates with the materials' hardness. We will use SGD to identify rules based on basic physical parameters which describe subsets of materials presenting a high bulk modulus. Thus, the bulk modulus is the target of our SGD analysis. The rules obtained by SGD using a training dataset of 504 materials will then be used to identify promising materials with a high bulk modulus from a pool of 12 096 candidate materials. Perovskites are a promising materials class^42,43^ for energy-related applications such as photovoltaics and catalysis^44–46^ and they have been the subject of a number of AI and machine-learning studies.^47–51^

The dataset^52^ used to train the SG rules contains 504 perovskites composed of *A* elements from the alkali, alkaline-earth, and scandium groups and lanthanides. *B* elements include transition metals and main-group elements such as bismuth, antimony, and germanium. The choice of *A* and *B* elements reflects common elements reported in perovskites.^44–46^ We only consider the cubic, highly symmetric perovskite structure in our dataset, which is often only stable at high temperatures. Thus, our analysis focuses on diversity of the chemical elements entering the material rather than on the diversity of structures. However, it is straightforward to extend the SGD approach to other, less symmetric crystal structures. We used 24 features characterizing the perovskites (Table 1). Two of the features are properties of the solid perovskite materials (denoted S), the equilibrium lattice constant (*a*_0_) and the cohesive energy (*E*_0_). The equilibrium lattice constant is the only structural degree of freedom of the cubic structure. The cohesive energy corresponds to the energy required to atomize the materials' crystal. Ten of the features are atomic properties of free atoms of the elements *A* or *B* (denoted A), such as orbital radii, ionization potential, and electronegativity. Finally, we included two features that depend on the composition of the material (denoted C), the expected oxidation states of *A* and *B* elements in the compound (*n*_A_ and *n*_B_, respectively). The bulk modulus and the features (except the atomic numbers of *A* and *B*, *n*_A_ and *n*_B_) were calculated using density-functional theory (DFT) with the PBEsol exchange-correlation functional. The bulk modulus is evaluated by fitting the Birch–Murnaghan equation of state to a series of energies of the crystal calculated using structures that present slightly larger or smaller volumes than the equilibrium volume. Further calculation details are provided elsewhere.^52^ We note that some of the features in Table 1 are correlated with each other. This is not a limitation for SGD. However, the presence of correlated features might result in similar SGs defined by slightly different rules.

**Table 1:** Features used to characterize the *AB*O_3_ perovskites in the SGD analysis

Type	Name	Symbol	Unit
S^a^	Equilibrium lattice constant^d^	*a*_0_	Å
S^a^	Cohesive energy^d^^e^	*E*_0_	eV per atom
A^b^	Radii of the valence-s orbitals of the *A* and *B* neutral atoms^d^	*r*_s,*A*_ and *r*_s,*B*_	Å
A^b^	Radii of the valence-s orbitals of the *A* and *B* +1 cations^d^	*r* ^cat^_s,*A*_ and *r*^cat^_s,*B*_	Å
A^b^	Radii of the highest-occupied orbitals of *A* and *B* neutral atoms^d^	*r*_val,*A*_ and *r*_val,*B*_	Å
A^b^	Radii of the highest-occupied orbitals of the *A* and *B* +1 cations^d^	*r* ^cat^_val,*A*_ and *r*^cat^_val,*B*_	Å
A^b^	Electron affinity of the *A* and *B* atoms^d^	EA_*A*_ and EA_*B*_	eV
A^b^	Ionization potential of the *A* and *B* atoms^d^	IP_*A*_ and IP_*B*_	eV
A^b^	Electronegativity of the *A* and *B* atoms^d^	EN_*A*_ and EN_*B*_	eV
A^b^	Kohn–Sham single-particle eigenvalue of the highest-occupied orbital of the *A* and *B* atoms^d^	*ε*_H,*A*_ and *ε*_H,*B*_	eV
A^b^	Kohn–Sham single-particle eigenvalue of the lowest-unoccupied orbital of the *A* and *B* atoms^d^	*ε*_L,*A*_ and *ε*_L,*B*_	eV
A^b^	Atomic numbers of *A* and *B* elements	*Z*_*A*_ and *Z*_*B*_	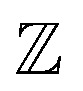
C^c^	Expected oxidation states of the elements *A* and *B* in the perovskite formula^f^	*n*_*A*_ and *n*_*B*_	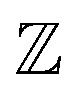

a Properties of the solid material.

b Properties of free atoms of elements constituting the material.

c Properties of the composition of the material.

d Evaluated using DFT-PBEsol.

e Energy needed per atom to atomize the crystal.

f Defined based on the periodic-table group of the *A* element and on the charge neutrality of the *AB*O_3_ composition, *i.e.*, *n*_*A*_ + *n*_*B*_ = 6.

#### 3.1.1 Collection of SG rules obtained with the positive-mean-shift utility function

We start by analyzing the results obtained with the positive-mean-shift utility function, defined as2
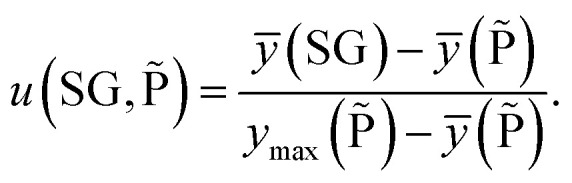
Here, *ȳ*(SG) and *ȳ*(P̃) are the mean values of the distribution of the target in the SG and in the entire dataset and *y*_max_(P̃) is the maximum value that the target assumes in the dataset. In our application, the target is the bulk modulus and y_max_(P̃) = 1.49 eV Å^−3^ for the ScMnO_3_ perovskite. The utility function in eqn (2) requests that the values of the target within the SG are high with respect to the mean value of the target in the dataset. It assumes that the distributions of target values in the SG and in the entire dataset are properly described by the mean values.

**Fig. 2:**
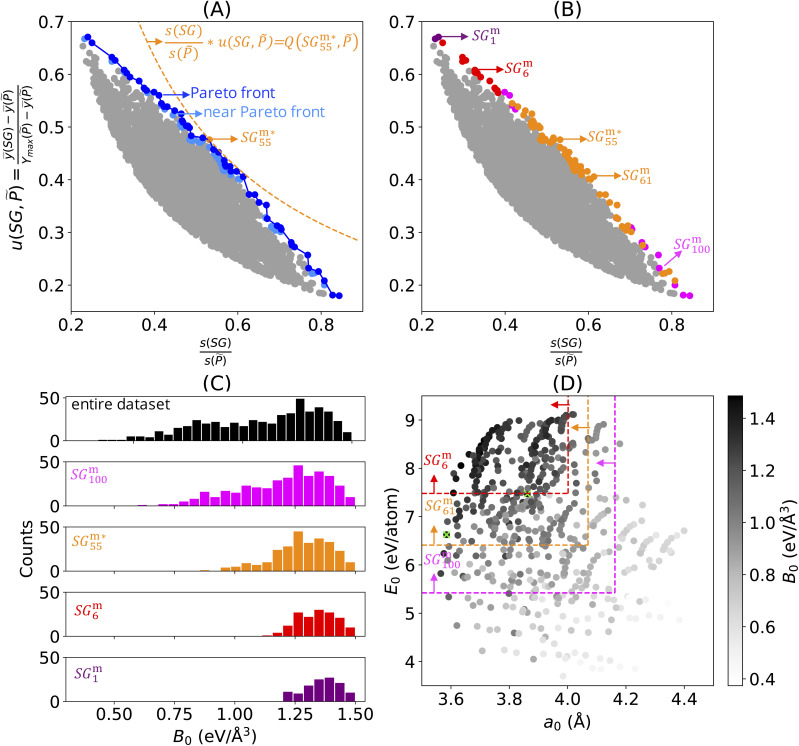
Collections of SGs describing perovskites with a high bulk modulus (*B*_0_) obtained using the multi-objective optimization approach and the positive-mean-shift utility function. The results for the full feature set containing the 24 features in Table 1 are shown. (A) The 5000 SGD solutions with high values of the objective function are shown in grey and the Pareto region of SGD solutions is shown in blue. The SG associated with the maximum value of the objective function is shown in orange. (B) The 109 SGs of the Pareto region are clustered according to their similarity *via* hierarchical clustering. Each cluster is shown in a different color. (C) Distributions of *B*_0_ in the entire training dataset and in some examples of SGs of the Pareto region. The rules associated with these SGs are shown in Table 2. (D) SG rules for some examples of SGs of the Pareto region that constrain the values of the equilibrium lattice constant (*a*_0_) and cohesive energy (*E*_0_).

The 5000 SGs with the highest objective-function values identified in the analysis using the positive-mean-shift utility function are shown as grey points in Fig. 2(A). The Pareto region is shown in blue in this plot: the 60 and 49 SGs belonging to the Pareto front and to the near-Pareto-front region are displayed in dark and light blue, respectively. This plot shows, in orange, the SG that maximizes the objective function, denoted as 
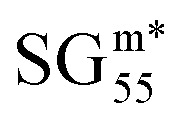
. The curve corresponding to the constant value of 
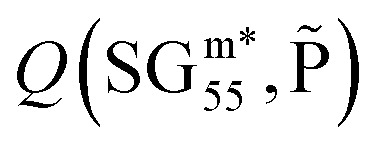
 is shown as a dashed orange line. Note that we assign the *i* indices to the SGs of the Pareto region SG^m^_*i*_ according to increasing values of relative SG size. The star in 
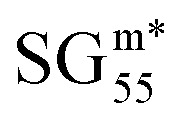
 indicates that this is the SG associated with the maximum objective-function value. The Pareto region contains many SGs with objective-function values close to the maximum at relative SG sizes in the range [0.4, 0.6]. Conversely, SGs in the Pareto region with relative sizes lower than 0.4 and higher than 0.6 present relatively lower objective-function values compared to the maximum value.

The Pareto-region concept leads to the identification of not one, but a collection of 109 SGs presenting multiple generality-exceptionality tradeoffs. However, it is unclear how to choose which of these SGs should be considered for a detailed analysis of physical insights or for materials discovery in larger candidate materials spaces. Many of the SGs of the Pareto region might be similar to each other and they might contain redundant information. In order to assess the variability of the SG rules of the Pareto region and to facilitate further analysis of these rules, we established a measure of similarity between SGs and used it to identify clusters of SGs containing similar SGs.^53^ This analysis is described in detail in the ESI.† In summary, the similarity is assessed using Jaccard indices. These indices consider that the similarity between two SGs is proportional to the overlap of their elements, *i.e.*, to the number of data points that satisfy the rules defining both SGs. Thus, this similarity will be high between SG rules that result in a similar selection of materials, even though the rules themselves might be different, *e.g.*, due to correlated key features or due to different thresholds. To obtain the clusters, we applied agglomerative hierarchical clustering.^54^

The results of the similarity analysis and clustering of the Pareto region in Fig. 2(A) for a chosen number of four clusters are displayed in Fig. 2(B). In this figure, the four identified clusters are displayed in four different colors. In general, the clusters of SGs correspond to different ranges of relative sizes. The cluster shown in orange, for instance, can be related to the SGD solutions with objective-function values close to the maximum in the relative size range of [0.4, 0.6]. Interestingly, a small cluster containing only three SGs is identified at low relative sizes. This indicates that these three SG rules are unique compared to the remaining ones. We note that the SGs of the Pareto region are spread in a continuous manner in the utility-function *vs.* relative-size plot in Fig. 2(A). The aim of the clustering technique is not to identify clusters of SGs that preexist in the utility-function *vs.* relative-size space, but rather to partition the pool of SGs of the Pareto region into clusters containing similar rules. This partitioning aims to facilitate the analysis of the many SGD solutions identified with the multi-objective-optimization approach.

We analyzed in more detail one SG per identified cluster. SG^m^_1_, SG^m^_6_, 
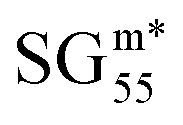
, and SG^m^_100_ are examples of SGs belonging to the purple, red, orange, and magenta clusters, respectively. The distributions of bulk-modulus values in the entire dataset and in the mentioned SGs are shown in Fig. 2(C). As the relative SG size decreases, the SGs of the Pareto region have higher mean bulk-modulus values and narrower bulk-modulus distributions. For the goal of identifying perovskites with an extremely high bulk modulus, the rules associated with SGs with low relative SG size and high mean bulk-modulus values, *e.g.*, associated with SG^m^_1_ or SG^m^_6_, are useful, since they provide a more focused description. Such SGs would not be detected based solely on the maximization of the objective function.

Next, we analyzed the rules defining SG^m^_1_, SG^m^_6_, 
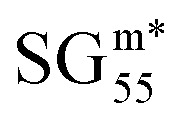
, and SG^m^_100_, shown in Table 2. The rules constrain the values of 3 key features, out of the 24 offered features: the equilibrium lattice constant (*a*_0_), cohesive energy (*E*_0_), and the radius of valence-s orbitals of +1 cations (cat) of the *B* element (*r*^cat^_s,*B*_). In particular, the *a*_0_ and *E*_0_ values are always constrained to maximum and minimum thresholds, respectively. Thus, perovskites with a short lattice constant and high cohesive energy tend to present a high bulk modulus. This reflects the inverse relationship of the bulk modulus with the lattice constant and the direct relationship of the bulk modulus with cohesive energy.^52^ This analysis illustrates how physical insights can be obtained from the key features identified by SGD.

**Table 2:** Characteristics of some of the SGs identified with the Pareto-region approach. Information on all other SGs of the Pareto region is provided in the ESI

Features	Index^b^	*Q*(SG, P̃)	*u*(SG, P̃)	*s*(SG)/*s*(P̃)	*ȳ*(SG)^c^	*y*_std_(SG)^d^	Rules
S, A, and C^a^	SG^m^_1_	0.16	0.67	0.24	1.36	0.07	*E*_0_ > 7.48 eV per atom ∧ *r*^cat^_s,*B*_ ≤ 1.44 Å
S, A, and C^a^	SG^m^_6_	0.19	0.63	0.31	1.34	0.08	*E*_0_ > 7.48 eV per atom ∧ *a*_0_ ≤ 4.00 Å
S, A, and C^a^	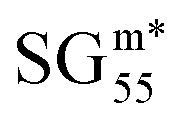	0.25	0.53	0.48	1.28	0.12	*E*_0_ ≥ 6.41 eV per atom ∧ *a*_0_ ≤ 4.07 Å ∧ *r*^cat^_s,*B*_ ≥ 0.94 Å
S, A, and C^a^	SG^m^_61_	0.25	0.45	0.55	1.27	0.13	*E*_0_ ≥ 6.41 eV per atom ∧ *a*_0_ ≤ 4.07 Å
S, A, and C^a^	SG^m^_100_	0.20	0.26	0.77	1.19	0.18	*E*_0_ ≥ 5.42 eV per atom ∧ *a*_0_ ≤ 4.16 Å
S, A, and C^a^	SG^JS^_5_	0.06	0.61	0.10	1.42	0.04	−4.55 ≤ *ε*_L,*B*_ < −4.33 eV ∧ *a*_0_ < 3.85 Å ∧ *n*_*B*_ < 3.5
S, A, and C^a^	SG^JS^_96_	0.10	0.45	0.22	1.37	0.06	*r*_val,*A*_ ≤ 1.51 Å ∧ EA_*B*_ ≥ −1.84 eV ∧ *r*^cat^_s,*B*_ ≤ 1.50 Å ∧ *r*_val,*B*_ ≤ 1.14 Å ∧ *E*_0_ > 7.11 eV per atom
S, A, and C^a^	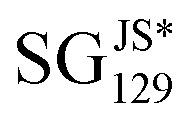	0.11	0.29	0.39	1.32	0.09	EA_*B*_ ≤ 0.31 eV ∧ *ε*_L,*B*_ ≤ −3.50 eV ∧ *r*^cat^_s,*B*_ ≥ 1.09 Å ∧ *E*_0_ ≥ 6.77 eV per atom
S, A, and C^a^	SG^JS^_169_	0.09	0.16	0.55	1.27	0.13	*E*_0_ ≥ 6.41 eV per atom ∧ *a*_0_ ≤ 4.07 Å
A and C^a^	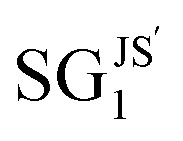	0.05	0.67	0.08	1.43	0.03	EA_*B*_ ≥ −1.03 eV ∧ −4.55 ≤ *ε*_L,*B*_ < −4.33 eV ∧ *n*_*B*_ < 4
A and C^a^	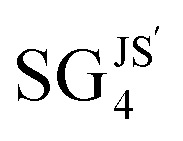	0.05	0.67	0.08	1.43	0.04	IP_*B*_ ≤ 7.82 eV ∧ *ε*_L,*B*_ < −4.33 eV ∧ *r*_val,*B*_ < 0.68 Å ∧ *n*_*B*_ < 3.5
A and C^a^	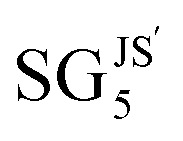	0.06	0.65	0.09	1.42	0.04	*r*_val,*A*_ < 1.28 Å ∧ EA_*B*_ ≤ 0.31 eV ∧ *Z*_*B*_ < 36 ∧ *r*_s,*B*_ ≥ 1.26 Å
A and C^a^	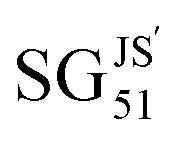	0.10	0.43	0.22	1.37	0.06	−5.11 ≤ *ε*_L,*B*_ ≤ −3.50 eV ∧ *r*^cat^_val,*B*_ ≤ 0.94 Å ∧ *n*_*A*_ > 2
A and C^a^	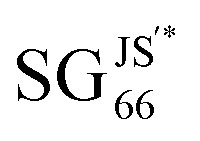	0.10	0.35	0.30	1.34	0.08	*r* ^cat^_val,*A*_ ≤ 1.38 Å ∧ *ε*_L,*B*_ ≤ −3.49 eV ∧ *r*_val,*B*_ ≤ 1.14 Å ∧ *n*_*A*_ ≥ 1.5
A and C^a^	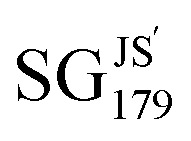	0.06	0.12	0.52	1.26	0.15	EN_*A*_ ≥ 2.76 eV ∧ EN_*B*_ ≤ 4.85 eV ∧ *r*_s,*B*_ ≥ 1.09 Å

a S, A, and C correspond to solid, atomic, and compositional, respectively (see Table 1).

b The star indicates the SG with the maximum value of objective (quality) function *Q* obtained with a given utility function and feature set. The superscripts “m” and “JS” correspond to the utility functions positive mean shift and cumulative Jensen–Shannon divergence, respectively.

c Mean value of the target within the SG, in eV Å^−3^.

d Standard deviation of the target within the SG, in eV Å^−3^.

The rules associated with SG^m^_6_ and SG^m^_100_ are presented in the coordinates of the key parameters *a*_0_ and *E*_0_ in Fig. 2(D). We also present, in this figure, the rules associated with SG^m^_61_, as an example of an SG that belongs to the orange cluster in Fig. 2(B) whose rules only depend on *a*_0_ and *E*_0_ – see Table 2. In this plot, the bulk-modulus values are indicated by the grey scale color of the circles. The figure shows graphically that a more focused description is achieved as the utility-function values increase (and the SG size decreases) within the Pareto region. This figure also highlights that more focused rules might arise at the expense of missing some high-bulk-modulus materials. For instance, some dark-grey circles corresponding to high-bulk-modulus materials are outside the limits of SG^m^_6_. Thus, these high-bulk-modulus materials are not captured by SG^m^_6_.

To understand which high-bulk-modulus materials might be “missed” in SG^m^_6_ as an example of an SG with a high utility function, we verified whether the 5% materials with the highest bulk moduli of the dataset are contained in SG^m^_6_. These are 26 perovskites presenting bulk moduli higher than 1.43 eV Å^−3^ and composed of the *B* elements chromium, manganese, iron, cobalt, and tungsten, and the *A* elements scandium, praseodymium, neodymium, cerium, promethium, yttrium, samarium, and beryllium. 24 of these 26 materials satisfy the rules associated with SG^m^_6_. The two high-bulk-modulus materials that do not satisfy the rules of SG^m^_6_ are BeMnO_3_ and BeWO_3_, with bulk moduli of 1.43 and 1.45 eV Å^−3^, respectively. These two materials present the lowest cohesive energies (6.63 and 7.47 eV per atom, respectively) among the 26 materials. They are shown as lime crosses in Fig. 2(D). Thus, they do not satisfy the inequality *E*_0_ > 7.48 eV per atom, which is part of the rules of SG^m^_6_ (Table 2). The bulk modulus for these two materials could be governed by a different mechanism compared to the materials that are part of SG^m^_6_. We will analyze this unexpected pattern in more detail in a dedicated subsection of the manuscript (see below).

We evaluated the variability of the identified SGs with respect to the dataset size by training the SGD with random selections of 75%, 50%, and 25% of the dataset. Even though the similarity between the SG identified based on the entire dataset and the SG identified based on a fraction of the dataset (measured using the Jaccard similarity index) decreases with decreasing data-set size, the SGs obtained with only 25% of the dataset and presenting large relative sizes are significantly similar compared to the SGs obtained with the entire dataset. The SGs obtained with 25% of the dataset and presenting low relative sizes, however, are significantly different from the SGs obtained with such relative sizes using the entire dataset. Thus, for the problem under consideration, SGD is efficient with up to 50% less data. More details of this analysis can be found in the ESI.†

#### 3.1.2 Collection of SG rules obtained with the cumulative-Jensen–Shannon-divergence utility function

We now turn our attention to the results obtained with a utility function based on the Jensen–Shannon divergence. The information-theoretic Jensen–Shannon divergence (*D*_JS_) is a symmetrized version of the Kullback–Leibler divergence (*D*_KL_), also known as relative entropy. The Jensen–Shannon divergence between the discrete distributions *R* and *S* is defined as3
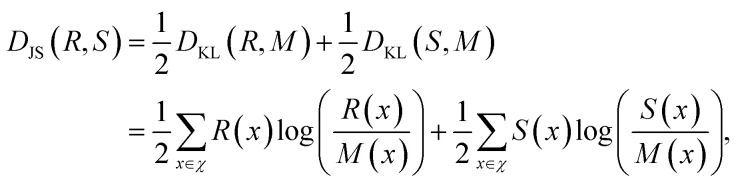
where 
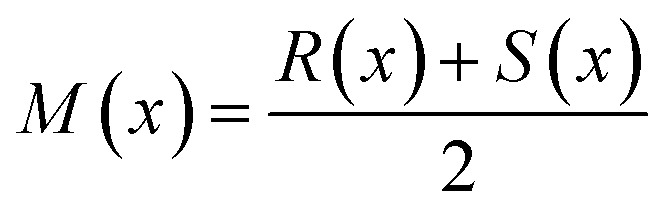
, and *χ* indicates the sample space. The Jensen–Shannon divergence measures the dissimilarity between two distributions. It assumes small values for similar distributions and increases as the distributions are shifted with respect to each other. The value of the Jensen–Shannon divergence also increases if two distributions have different narrownesses. In our SGD analysis, the cumulative formulation of the Jensen–Shannon divergence,^41^ denoted *D*_cJS_, is used as the utility function. The divergence is evaluated between the distribution of the target values in the SG and the distribution of the target values in the entire dataset. This utility function favors the selection of SGs presenting distributions of target values that are shifted and narrower compared to the distribution of the entire dataset. However, it does not explicitly require the values of the target in the SG to be high or low. Moreover, the cumulative Jensen–Shannon divergence does not make assumptions on the shape of the distributions. Thus, it can handle distributions that deviate significantly from a Gaussian more efficiently than utility functions based on the mean shift. We stress that other measures of similarity between distributions such as the Bhattacharyya distance can be used as utility functions in SGD.

**Fig. 3:**
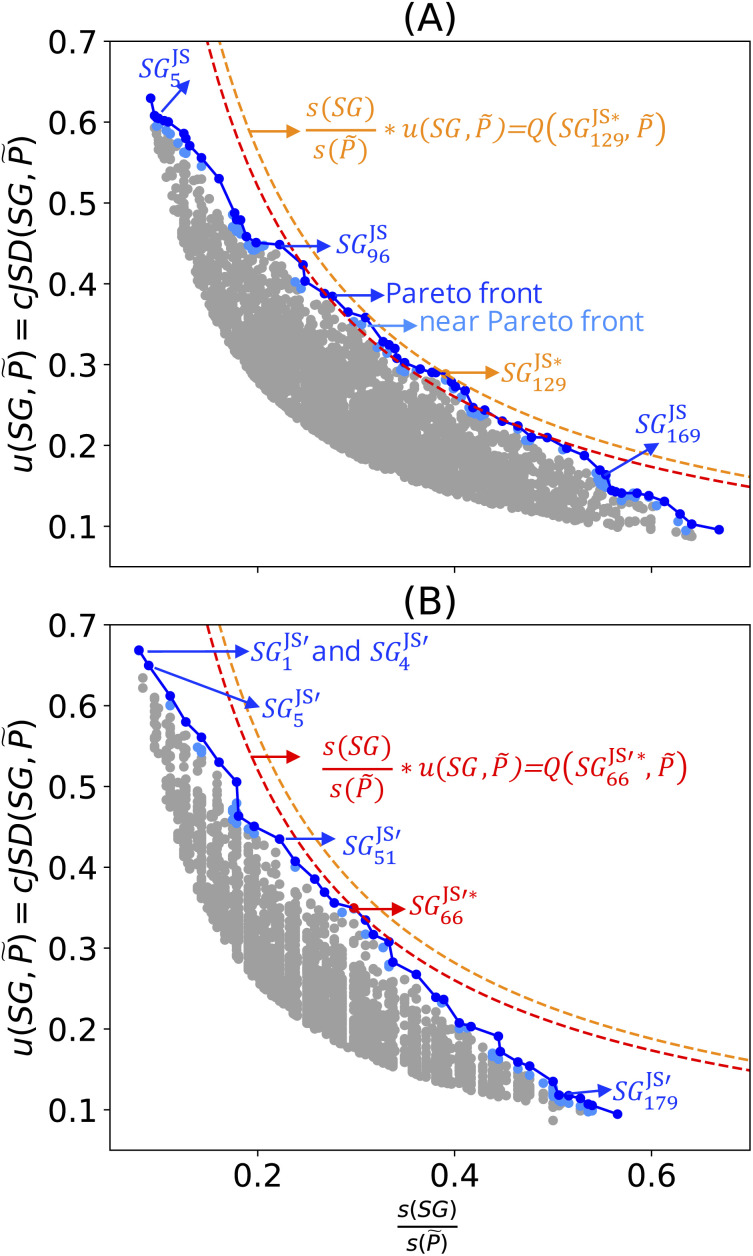
Collections of SGs describing perovskites with a high bulk modulus using the cumulative formulation of the Jensen–Shannon divergence as the utility function. The 5000 SGD solutions with high values of the objective function are shown in grey and the Pareto region is displayed in blue. The SG associated with the maximum value of the objective function is shown in orange or red. The two panels show the results for two different sets of features. (A) Results for the full feature set containing the 24 features in Table 1. (B) Results for the reduced feature set containing 22 atomic and compositional features (see Table 1). The rules associated with the SGs indicated in the figure are shown in Table 2.

The results obtained with the cumulative Jensen–Shannon-divergence utility function and with the full set of the 24 features are shown in Fig. 3(A) and in Table 2. The Pareto front and region contain 101 and 189 SGs, respectively. The SGs identified in the Pareto region contain materials with a high bulk modulus and they present narrow distributions of target values. We analyzed the selectors defining some of the SGs of this Pareto region. The rules associated with SG^JS^_5_, SG^JS^_96_, 
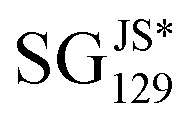
, and SG^JS^_169_ constrain the values of the key features: the equilibrium lattice constant (*a*_0_), cohesive energy (*E*_0_), the radius of valence-s orbitals of +1 cations (cat) of the *B* element (*r*^cat^_s,*B*_), the Kohn–Sham single-particle eigenvalue of the lowest-unoccupied orbital of the *B* atom (*ε*_L,*B*_), the expected oxidation state of *B* in the perovskite (*n*_*B*_), the radius of the highest-occupied orbital of *A* and *B* neutral atoms (*r*_val,*A*_ and *r*_val,*B*_, respectively), and the electron affinity of the element *B* (EA_*B*_). Therefore, the rules highlight that the lattice constant and the cohesive energy are important parameters for describing high-bulk-modulus materials, along with atomic and compositional properties that mainly reflect the nature of the *B* element of the *AB*O_3_ perovskites.

Let us now compare the results obtained with the positive-mean-shift with the results obtained with the cumulative-Jensen–Shannon-divergence utility functions. The Pareto region of SGs identified based on the positive-mean-shift utility function is associated with relative SG sizes in the approximate range [0.20, 0.80] (Fig. 2(A)). The range of relative SG sizes in the Pareto region identified for the case of the cumulative-Jensen–Shannon-divergence utility function is, in turn, *ca.* [0.10, 0.70] (Fig. 3(A)). Thus, optimal SGs with relative SG sizes below 0.20, *i.e.*, SGs that contain less than 20% of the dataset, could only be obtained with the utility function based on the Jensen–Shannon divergence. These SGs with small size are associated with distributions of bulk-modulus values that are narrower and more shifted towards high values than those associated with the SGs identified with the positive-mean-shift utility function. For instance, the materials selected in SG^JS^_5_ and SG^JS^_96_ present standard deviations of bulk-modulus values of 0.04 and 0.06 eV Å^−3^, respectively. The mean values of the bulk modulus among the perovskites in these two SGs are 1.42 and 1.37 eV Å^−3^, respectively. None of the SGs identified with the positive mean shift present higher mean values or lower standard deviation values (see Table 2). Thus, the rules corresponding to small SGs identified with the cumulative Jensen–Shannon divergence are more focused on the high bulk modulus. This can be related to the fact that only this utility function explicitly favors narrow SGs.

The SG rules identified using the cumulative-Jensen–Shannon-divergence utility function contain, in general, more statements and more features compared to the SG rules identified using the positive mean shift (Table 2). However, we note that the equilibrium lattice constant, the cohesive energy, and the radii of *B* atoms are identified as key features by both approaches. The cumulative Jensen–Shannon-divergence utility function provides more focused rules for the present dataset and it is thus better than the positive-mean-shift for the purpose of identifying exceptional perovskites with very high bulk moduli. However, we stress that this utility function does not explicitly require low or high values of the target. This is a disadvantage compared to the positive-mean-shift utility function. Dispersion-corrected utility functions simultaneously take into account positive or negative shifts of the mean (or of the medians) of target values and the narrowness of the distributions of targets in the SG. These utility functions were proposed in order to simultaneously incorporate the requirements for a shift in a specific direction and for small narrowness.^31^

#### 3.1.3 SG rules obtained with a reduced set of features

So far, we have used the entire set of the 24 features in Table 1 to obtain SGs of perovskites with a high bulk modulus. SGD identified the equilibrium lattice constant (*a*_0_) and the cohesive energy (*E*_0_) among the key features required for describing high-bulk-modulus materials. However, in order to calculate *a*_0_ and *E*_0_ using DFT, the geometry of the materials needs to be optimized. This optimization corresponds to the majority of the work needed to calculate the bulk modulus itself. Thus, from the standpoint of exploring a large materials space, *a*_0_ and *E*_0_ are impractical (expensive) features, since one needs to evaluate these quantities for the materials under consideration in order to apply the SG rules. In order to obtain SG rules that describe high-bulk-modulus perovskites based on easily accessible features, we have also considered a reduced set of 22 features by excluding *a*_0_ and *E*_0_. Thus, we only considered the atomic and compositional features. In addition to this crucial cost aspect, this analysis also illustrates how the SG rules change when the feature set changes and, in particular, when important features are not included in SGD. The identification of appropriate rules based solely on atomic and compositional features is a remarkable challenge for SGD, since the relationship between these basic features and the bulk modulus is significantly more indirect compared to the relationship between the bulk modulus and *a*_0_ or *E*_0_.^52^

We identified the Pareto region of SGs using the reduced set of 22 features and the cumulative Jensen–Shannon-divergence utility function. The SGs identified with this approach are denoted as 
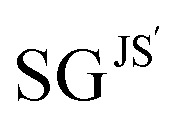
, where ′ indicates the reduced feature set. The results are shown in Fig. 3(B) and in Table 2. The identified Pareto front and near the Pareto front contain 66 and 136 SGs, respectively. The maximum value of the objective function obtained with the reduced feature set is slightly lower than that obtained with the full feature set (0.10 and 0.11, respectively, see orange and red dashed lines in Fig. 3(A) and (B)). This indicates that the quality of the SG description is lower with the reduced feature set. The SG identified based on the reduced set of features that displays the maximum objective function, denoted as 
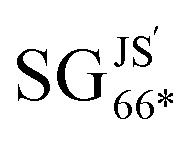
 in Table 2 and Fig. 3(B), contains 150 materials. This SG contains fewer materials than the SG identified with all the features SG^JS*^_129_ (197 materials). However, the overlap between the two SGs is significant. Indeed, 125 materials are present in both SGs. Thus, there is a high similarity between both descriptions.

SG rules focusing on high-bulk-modulus materials in the low relative size portion of the Pareto region were also obtained with this reduced feature set. For instance, 
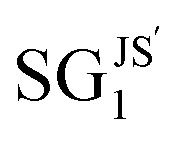
, 
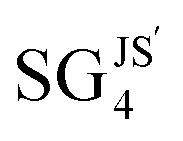
, and 
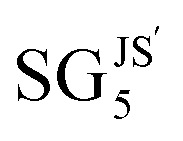
 display mean bulk-modulus values of 1.43, 1.43, and 1.42 eV Å^−3^ and standard deviations of 0.03, 0.04 and 0.04 eV Å^−3^, respectively. These figures are similar to those associated with SG^JS^_5_, which was identified based on the entire set of the 24 features. The SGs 
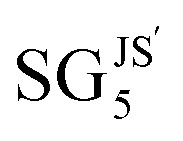
 and SG^JS^_5_ contain, respectively, 45 and 48 materials. 40 materials are present in both SGs. This reflects a significant similarity between both descriptions. Thus, the SGs with small relative size identified in the Pareto region with the reduced set of features are comparable to those obtained with the full feature set. The rules associated with the SGs identified using the reduced set of 22 features depend on some key parameters that were also identified by the analysis of the entire set of the 24 features and the cumulative-Jensen–Shannon-divergence utility function, *e.g.*, radii of *A* and *B* elements, Kohn–Sham single-particle eigenvalue of the lowest-unoccupied orbital of the *B* atom (*ε*_L,*B*_), electron affinity of the *B* atom (EN_*B*_), and expected oxidation state of the *B* element in the perovskite. However, some additional key features are identified in the analysis of the reduced set of features, such as the ionization potential of the *B* atom (IP_*B*_), the atomic charge of the *B* element (*Z*_*B*_), the expected oxidation state of the *A* element in the perovskite (*n*_*A*_), and the electronegativity of the *A* element (EN_*A*_). This shows how SGD attempts to reconstruct the information contained in the important features *a*_0_ and *E*_0_ by using the information on other offered features. Overall, the results obtained with the reduced feature set are comparable to those obtained with all 24 features. The rules derived based on the reduced feature set can thus be applied to identify high-bulk-modulus materials in larger materials spaces compared to the training set.

#### 3.1.4 Exploitation of the SG rules for the identification of perovskites with a high bulk modulus

We applied the SG rules trained on 504 single ABO_3_ perovskites with the reduced set of 22 features to identify materials with a high bulk modulus (*B*_0_) out of a candidate materials space of 12 096 compounds. This candidate material space was created by considering additional *A* and *B* elements that were not included in the training set (*A*: thorium and protactinium; *B*: hafnium, rhenium, osmium, iridium, gold, mercury, and thallium). Additionally, we combined two different *B* elements to form double perovskites with the formula *A*_2_*BB*′O_6_. In the case of the double perovskites, the features related to the *B* element are defined as the (composition) average of the features associated with the two different *B* and *B*′ elements. In this analysis, we are explicitly considering a finite set of *A*_2_*BB*′O_6_ materials that contain a 1 : 1 *B* : *B*′ stoichiometric ratio. However, the material space of double perovskites is practically infinite, since any proportion of *B* and *B*′ is possible, *i.e.*, any 
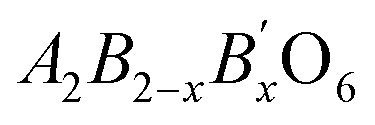
 formula with *x* in the range [0.0, 2.0] corresponds to a material in this space. In order to assess the usefulness of SG rules identified with the Pareto-region approach with respect to the SG rules associated with the maximum value of the objective function, we applied two different sets of SG rules to select materials from the candidate materials space that likely present high bulk moduli. The first set of rules corresponds to 
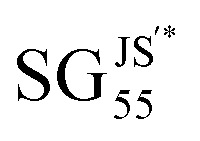
, which are associated with the SG with maximum value of the objective function. These rules are satisfied by 4518 of the 12 096 candidates. The second set of rules corresponds to 
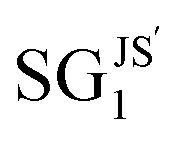
, 
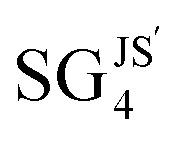
, and 
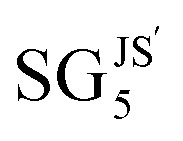
, which are associated with the SGs in the Pareto region with high utility-function values, *i.e.*, high exceptionality. These rules are satisfied by 238 materials, out of the 12 096 candidates, *i.e.*, 1.97% of the candidate materials space. Then, we randomly selected 50 of the 4518 selected materials and 50 of the 238 selected materials and evaluated their *B*_0_ using DFT-PBEsol calculations. For comparison, we have also calculated the *B*_0_ of 50 perovskites that were randomly selected from the 12 096 materials.

**Fig. 4:**
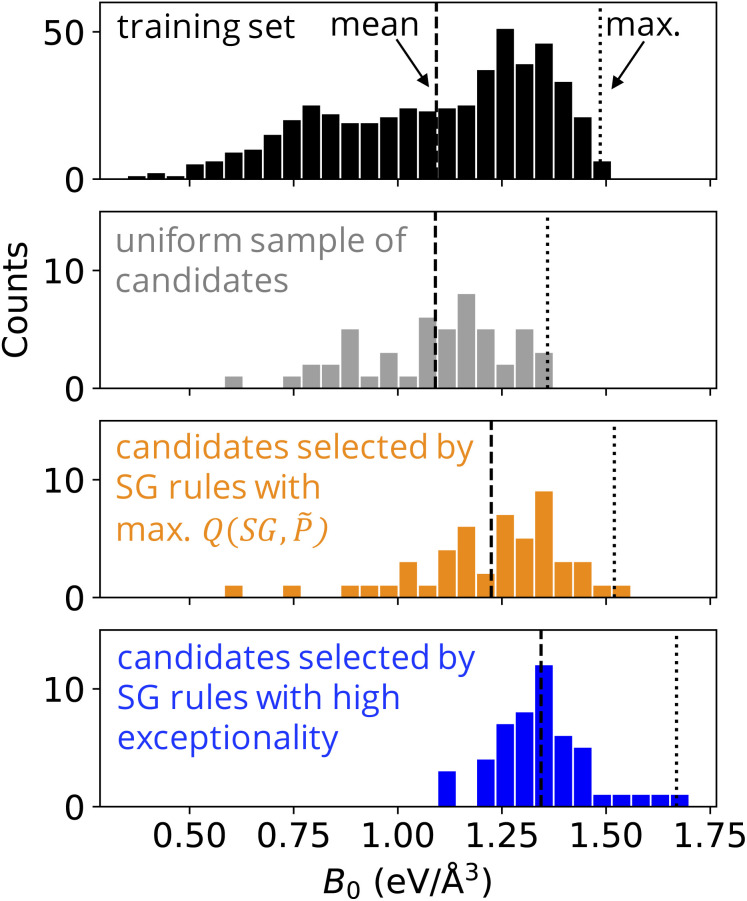
The SG rules describing perovskites with a high bulk modulus (*B*_0_) trained on 504 single *AB*O_3_ perovskites are applied to identify promising single *AB*O_3_ and double *A*_2_*BB*′O_6_ perovskites from a candidate space containing 12 096 materials. The histograms show the distribution of *B*_0_ among the materials of the training dataset (in black), among 50 materials randomly selected from the candidate space (in grey), among 50 materials of the candidate space selected according to the SG rules 
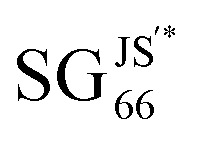
 (in orange), and among 50 materials of the candidate space suggested by the SG rules 
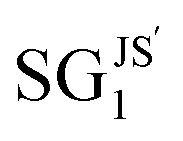
, 
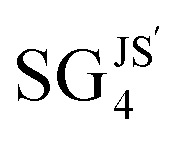
, and 
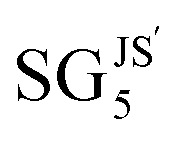
 (in blue). The dashed and dotted lines indicate the mean and maximum (max.) *B*_0_ values of each distribution, respectively.

The distribution of *B*_0_ for the materials that were randomly selected from the candidate materials space (Fig. 4, in grey) has practically the same mean value compared to that of the distribution of *B*_0_ in the training set (Fig. 4, in black), *i.e.*, 1.09 eV Å^−3^. This indicates that the training data might be representative of this specific candidate materials space. The highest *B*_0_ among the materials randomly selected from the candidate materials space is 1.36 eV Å^−3^. This value is significantly lower than the highest *B*_0_ in the training dataset, 1.49 eV Å^−3^ for ScMnO_3_.

The distribution of *B*_0_ for the materials that were selected from the candidate materials space using the SG rules 
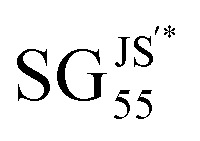
 (Fig. 4, in orange) is concentrated in high *B*_0_, with a mean *B*_0_ value of 1.22 eV Å^−3^. One material suggested by these SG rules has *B*_0_ higher than the highest value in the training dataset, PaCoOsO_6_, with a bulk modulus of 1.52 eV Å^−3^, respectively. This value is slightly higher than the highest *B*_0_ in the training dataset (1.49 eV Å^−3^). However, we note that the rules suggested several materials that turned out to have relatively low values of *B*_0_.

The distribution of *B*_0_ for the materials that were selected from the candidate materials space using the SG rules 
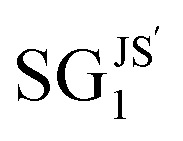
, 
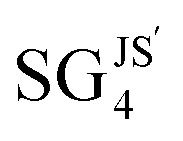
, and 
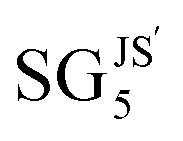
 (Fig. 4, in blue) is concentrated in higher values compared to the other distributions, with a mean *B*_0_ value of 1.35 eV Å^−3^. Additionally, four of the materials suggested by the SG rules have *B*_0_ higher than the highest value in the training dataset. These are PaMnO_3_, Pa_2_CrFeO_6_, Pa_2_VCrO_6_, and PaVO_3_, with a bulk modulus of 1.67, 1.63, 1.59, and 1.55 eV Å^−3^, respectively. Thus, the SG rules lead to the identification of materials with *B*_0_ up to 13% higher than any observation in the training dataset. This result indicates that the SGs identified by the Pareto-region analysis can point at more exceptional materials compared to the standard SGD approach. In particular, in the present use case, materials that present higher performance than the known compounds of the training set were more efficiently selected using the focused SG rules provided by the multi-objective optimization of SGs. We note that in previous studies SGD was compared with global approaches such as decision trees in the context of materials science applications.^34,55^ The results indicate that SGD is more efficient in describing exceptional situations compared to the decision-tree approach. This can be related to the fact that the objective function of the decision tree aims at a good performance on average, while the objective function of SGD eqn (1) can focus on statistically exceptional situations.

The dataset utilized to train SGD and the verification of SGD suggestions are based on DFT-PBEsol calculations. Even though DFT-PBEsol presents an overall good accuracy for describing properties of solid materials such as the bulk modulus,^56^ the errors of DFT-PBEsol should be taken into account when comparing the reported bulk moduli with experimental results.

#### 3.1.5 Investigation of high-bulk-modulus perovskites that are not captured using the SG rules

When analyzing the SG rules, we highlighted that BeMnO_3_ and BeWO_3_ present bulk moduli above the 95%-ile of the bulk-modulus distribution in the training set (1.43 and 1.45 eV Å^−3^, respectively) but they are not contained in the high-utility-function SG SG^m^_6_ identified in Fig. 2. In the perovskite structure, the *A* cations are larger than the *B* cations and the relationships between the radii of the *A* and *B* cations and the anions determine the thermodynamic stability of the perovskite structure, as given by the tolerance factors.^48,57^ Beryllium and magnesium are the only two *A* elements in our dataset for which the radius of *A* (*e.g.*, *r*_s,*A*_) might be smaller than the radius of *B* (*e.g.*, *r*_s,*B*_). This is the case for the materials BeMnO_3_ and BeWO_3_. Indeed, *r*_s,Be_ < *r*_s,Mn_ and *r*_s,Be_ < *r*_s,W_. Thus, beryllium would most likely occupy the *B* site of these cubic perovskite structures. Indeed, several reports discuss the properties of perovskites composed of beryllium at the *B* sites, *i.e.*, coordinated with 6 oxygen or halide anions.^58–60^ This observation motivated us to evaluate the bulk modulus of the perovskites MnBeO_3_ and WBeO_3_, where beryllium sits at the *B* site and manganese or tungsten sits at the *A* sites. The calculated bulk moduli are equal to 1.44 and 1.82 eV Å^−3^. The bulk modulus of WBeO_3_ is 22% higher than the highest value in the training set and is the highest bulk modulus identified in this paper. We have also evaluated the bulk modulus of perovskites with *B* = Be and other transition metals of the third row of the periodic table as *A* elements, namely hafnium, tantalum, rhenium, osmium and iridium. The bulk modulus of the materials HfBeO_3_, TaBeO_3_, ReBeO_3_, OsBeO_3_, and IrBeO_3_ are equal to 1.70, 1.81, 1.75, 1.63, and 1.57 eV Å^−3^, respectively. These values are also relatively high compared to the values of the training set and they show that several beryllium based materials might be exceptional. This analysis illustrates how the exploratory nature of SGD analysis can identify unexpected patterns and anomalies, which might lead in turn to the identification of exceptional materials.

We note that the rules identified with SGD will be valid as long as the physical processes governing the materials in the training dataset also govern the behavior of the materials in the materials space to be explored. This is a crucial aspect, since the choice of the materials in the training dataset is often influenced by bias and the number of materials in the training set is very small compared to the practically infinite space of possible materials. In order to cover portions of the materials space where underlying processes different from those present in the training set are important, the incorporation of new data points and retraining of SG rules will be required. Indeed, exceptional SGs and phenomena might emerge in regions of the data space that are not sufficiently covered by the training dataset. Additionally, the identified SGs can be associated with genuinely exceptional phenomena, but they might also correspond to measurement artifacts when the data are generated by an experiment or calculation subjected to noise.^61^ These two situations would not be distinguished by SGD. However, by analyzing the SG rules and key identified parameters, one might be able to judge whether SGD identified correlations that have a physical meaning. For instance, the rules derived in Fig. 2(D) reflect that stronger bonds between atoms in the crystal result in short lattice constants, high cohesive energy, and a high bulk modulus. Additionally, SGD models for materials rely on the fact that the offered features correlate with the underlying physical processes governing the materials. Thus, the choice of features is critical. The performance of SGD can be assessed by cross-validation, as described in ref. 35. Finally, useful materials might present unusual combinations of different materials properties. These could also be considered exceptional materials. Identifying such materials calls for multi-objective optimization of materials properties.^62–65^ SGD can be adapted for this scenario and this aspect will be addressed in an upcoming contribution.

## 4 Conclusions

We introduced an approach for the identification of coherent collections of SGs of the “Pareto region” with respect to the SG size and exceptionality objectives of the SGD analysis. The concept was demonstrated by the learning of rules that describe perovskites with a high bulk modulus. Our results show that rules focused on exceptional materials do not necessarily correspond to the one SG that maximizes the objective function, but these rules can be identified with the Pareto-region concept. This analysis does not require additional computational effort, since the SGD solutions with high objective-function values are obtained on the fly during the optimization of the objective function. We used the SG rules obtained by the multi-objective approach to identify exceptional perovskites with the bulk modulus up to 13% higher than the highest value found in the training set of 504 materials, out of a materials space of more than 12 000 materials.

## Author contributions

L. F. conceived the project and performed the SGD analysis and DFT calculations. L. F. and M. S. wrote the manuscript jointly.

## Conflicts of interest

The authors declare no competing interests.

## Supplementary Material

DD-004-D5DD00174A-s001

## Data Availability

All input and output files of the DFT calculations and datasets are available in ref. 52 and 66. The SGD analysis is available at https://github.com/lfoppa/Multi-objective-optimization-of-subgroups.

## References

[cit1] Lewis N. S., Nocera D. G. (2006). Proc. Natl. Acad. Sci. U. S. A..

[cit2] Chu S., Majumdar A. (2012). Nature.

[cit3] Davies D., Butler K., Jackson A., Morris A., Frost J., Skelton J., Walsh A. (2016). Chem.

[cit4] Schrier J., Norquist A. J., Buonassisi T., Brgoch J. (2023). J. Am. Chem. Soc..

[cit5] Ramprasad R., Batra R., Pilania G., Mannodi-Kanakkithodi A., Kim C. (2017). npj Comput. Mater..

[cit6] Schmidt J., Marques M. R. G., Botti S., Marques M. A. L. (2019). npj Comput. Mater..

[cit7] Raabe D., Mianroodi J. R., Neugebauer J. (2023). Nat. Comput. Sci..

[cit8] Bauer S., Benner P., Bereau T., Blum V., Boley M., Carbogno C., Catlow C. R. A., Dehm G., Eibl S., Ernstorfer R., Fekete Á., Foppa L., Fratzl P., Freysoldt C., Gault B., Ghiringhelli L. M., Giri S. K., Gladyshev A., Goyal P., Hattrick-Simpers J., Kabalan L., Karpov P., Khorrami M. S., Koch C. T., Kokott S., Kosch T., Kowalec I., Kremer K., Leitherer A., Li Y., Liebscher C. H., Logsdail A. J., Lu Z., Luong F., Marek A., Merz F., Mianroodi J. R., Neugebauer J., Pei Z., Purcell T. A. R., Raabe D., Rampp M., Rossi M., Rost J.-M., Saal J., Saalmann U., Sasidhar K. N., Saxena A., Sbailò L., Scheidgen M., Schloz M., Schmidt D. F., Teshuva S., Trunschke A., Wei Y., Weikum G., Xian R. P., Yao Y., Yin J., Zhao M., Scheffler M. (2024). Modell. Simul. Mater. Sci. Eng..

[cit9] Foppa L., Ghiringhelli L. M., Girgsdies F., Hashagen M., Kube P., Hävecker M., Carey S. J., Tarasov A., Kraus P., Rosowski F., Schlögl R., Trunschke A., Scheffler M. (2021). MRS Bull..

[cit10] Meredig B., Antono E., Church C., Hutchinson M., Ling J., Paradiso S., Blaiszik B., Foster I., Gibbons B., Hattrick-Simpers J., Mehta A., Ward L. (2018). Mol. Syst. Des. Eng..

[cit11] Kauwe S. K., Graser J., Murdock R., Sparks T. D. (2020). Comput. Mater. Sci..

[cit12] Muckley E. S., Saal J. E., Meredig B., Roper C. S., Martin J. H. (2023). Digit. Discov..

[cit13] Li K., DeCost B., Choudhary K., Greenwood M., Hattrick-Simpers J. (2023). npj Comput. Mater..

[cit14] Li K., Rubungo A. N., Lei X., Persaud D., Choudhary K., DeCost B., Dieng A. B., Hattrick-Simpers J. (2025). Commun. Mater..

[cit15] Wang Y., Wagner N., Rondinelli J. M. (2019). MRS Commun..

[cit16] Shahriari B., Swersky K., Wang Z., Adams R. P., de Freitas N. (2016). Proc. IEEE.

[cit17] Zhang H., Chen W. W., Rondinelli J. M., Chen W. (2023). Appl. Phys. Rev..

[cit18] Siemenn A. E., Ren Z., Li Q., Buonassisi T. (2023). npj Comput. Mater..

[cit19] Biswas A., Liu Y., Creange N., Liu Y.-C., Jesse S., Yang J.-C., Kalinin S. V., Ziatdinov M. A., Vasudevan R. K. (2024). npj Comput. Mater..

[cit20] Hirschfeld L., Swanson K., Yang K., Barzilay R., Coley C. W. (2020). J. Chem. Inf. Model..

[cit21] Palmer G., Du S., Politowicz A., Emory J. P., Yang X., Gautam A., Gupta G., Li Z., Jacobs R., Morgan D. (2022). npj Comput. Mater..

[cit22] Tran K., Neiswanger W., Yoon J., Zhang Q., Xing E., Ulissi Z. W. (2020). Mach. Learn.: Sci. Technol..

[cit23] Borg C. K. H., Muckley E. S., Nyby C., Saal J. E., Ward L., Mehta A., Meredig B. (2023). Digit. Discov..

[cit24] Serraino G. and Uryasev S., in Conditional Value-at-Risk (CVaR), ed. S. I. Gass and M. C. Fu, Springer US, Boston, MA, 2013, pp. 258–266

[cit25] Seko A., Hayashi H., Kashima H., Tanaka I. (2018). Phys. Rev. Mater..

[cit26] Kuban M., Gabaj Š., Aggoune W., Vona C., Rigamonti S., Draxl C. (2022). MRS Bull..

[cit27] Jia H., Horton M., Wang Y., Zhang S., Persson K. A., Meng S., Liu M. (2022). Adv. Sci..

[cit28] Wrobel S. , European Conference on Principles of Data Mining and Knowledge Discovery, 1997

[cit29] Friedman J. H., Fischer N. I. (1999). Stat. Comput..

[cit30] Goldsmith B. R., Boley M., Vreeken J., Scheffler M., Ghiringhelli L. M. (2017). New J. Phys..

[cit31] Boley M., Goldsmith B. R., Ghiringhelli L. M., Vreeken J. (2017). Data Min. Knowl. Discovery.

[cit32] Sutton C., Boley M., Ghiringhelli L. M., Rupp M., Vreeken J., Scheffler M. (2020). Nat. Commun..

[cit33] Li H., Liu Y., Chen K., Margraf J. T., Li Y., Reuter K. (2021). ACS Catal..

[cit34] Foppa L., Ghiringhelli L. M. (2022). Top. Catal..

[cit35] Foppa L., Sutton C., Ghiringhelli L. M., De S., Löser P., Schunk S. A., Schäfer A., Scheffler M. (2022). ACS Catal..

[cit36] Novak P. K. , Lavrač N. and Webb G. I., in Supervised Descriptive Rule Induction, ed. C. Sammut and G. I. Webb, Springer US, Boston, MA, 2010, pp. 938–941

[cit37] Boley M. , Lucchese C., Paurat D. and Gärtner T., Proceedings of the 17th ACM SIGKDD International Conference on Knowledge Discovery and Data Mining, New York, NY, USA, 2011, pp. 582–590

[cit38] Boley M. , Moens S. and Gärtner T., Proceedings of the 18th ACM SIGKDD International Conference on Knowledge Discovery and Data Mining, New York, NY, USA, 2012, pp. 69–77

[cit39] Grosskreutz H. , Rüping S. and Wrobel S., Machine Learning and Knowledge Discovery in Databases, Berlin, Heidelberg, 2008, pp. 440–456

[cit40] Rivera-Arrieta H. I., Foppa L. (2025). ACS Catal..

[cit41] Nguyen H.-V. and Vreeken J., Machine Learning and Knowledge Discovery in Databases: European Conference, ECML PKDD 2015, Porto, Portugal, September 7-11, 2015, Proceedings, Part II 15, 2015, pp. 173–189

[cit42] Peña M. A., Fierro J. L. G. (2001). Chem. Rev..

[cit43] Travis W., Glover E. N. K., Bronstein H., Scanlon D. O., Palgrave R. G. (2016). Chem. Sci..

[cit44] Jena A. K., Kulkarni A., Miyasaka T. (2019). Chem. Rev..

[cit45] Hwang J., Rao R. R., Giordano L., Katayama Y., Yu Y., Shao-Horn Y. (2017). Science.

[cit46] Kim J. Y., Lee J.-W., Jung H. S., Shin H., Park N.-G. (2020). Chem. Rev..

[cit47] Pilania G., Mannodi-Kanakkithodi A., Uberuaga B. P., Ramprasad R., Gubernatis J. E., Lookman T. (2016). Sci. Rep..

[cit48] Bartel C. J., Sutton C., Goldsmith B. R., Ouyang R., Musgrave C. B., Ghiringhelli L. M., Scheffler M. (2019). Sci. Adv..

[cit49] Tao Q., Xu P., Li M., Lu W. (2021). npj Comput. Mater..

[cit50] Ihalage A., Hao Y. (2021). npj Comput. Mater..

[cit51] Gu G. H., Jang J., Noh J., Walsh A., Jung Y. (2022). npj Comput. Mater..

[cit52] Foppa L., Purcell T. A. R., Levchenko S. V., Scheffler M., Ghiringhelli L. M. (2022). Phys. Rev. Lett..

[cit53] Niemann U. , Spiliopoulou M., Preim B., Ittermann T. and Völzke H., 2017 IEEE 30th International Symposium on Computer-Based Medical Systems (CBMS), 2017, pp. 582–587

[cit54] Nielsen F. , in Hierarchical Clustering, Springer International Publishing, Cham, 2016, pp. 195–211

[cit55] Mazheika A., Wang Y.-G., Valero R., Viñes F., Illas F., Ghiringhelli L. M., Levchenko S. V., Scheffler M. (2022). Nat. Commun..

[cit56] Zhang G.-X., Reilly A. M., Tkatchenko A., Scheffler M. (2018). New J. Phys..

[cit57] Goldschmidt V. M. (1926). Naturwissenschaften.

[cit58] Li C., Lu X., Ding W., Feng L., Gao Y., Guo Z. (2008). Acta Crystallogr., Sect. B: Struct. Sci..

[cit59] Mahmood Q., Hassan M., Yaseen M., Laref A. (2019). Chem. Phys. Lett..

[cit60] Kumari P., Srivastava V., Sharma R., Kaur N., Ullah H. (2024). Mater. Today Commun..

[cit61] Harris S. B., Vasudevan R., Liu Y. (2025). npj Comput. Mater..

[cit62] del Rosario Z., Rupp M., Kim Y., Antono E., Ling J. (2020). J. Chem. Phys..

[cit63] Jablonka K. M., Jothiappan G. M., Wang S., Smit B., Yoo B. (2021). Nat. Commun..

[cit64] Shi B., Lookman T., Xue D. (2023). Mater. Genome Eng. Adv..

[cit65] Low A. K. Y., Mekki-Berrada F., Gupta A., Ostudin A., Xie J., Vissol-Gaudin E., Lim Y.-F., Li Q., Ong Y. S., Khan S. A., Hippalgaonkar K. (2024). npj Comput. Mater..

[cit66] Foppa L. , Purcell T., Levchenko S., Scheffler M. and Ghiringhelli L. M., 2022, 10.17172/NOMAD/2022.02.21-3 35960572

