# Supplementary material for: Coherent collections of rules describing exceptional materials identified with a multi-objective optimization of subgroups
Source: Digit Discov. 2025 Jun 25;4(8):2175–87. doi: 10.1039/d5dd00174a (PMC12257468; doi:10.1039/d5dd00174a)
Supplement: DD-004-D5DD00174A-s001 [file DD-004-D5DD00174A-s001.pdf]

Supplementary Information (SI) for Digital Discovery.
This journal is © The Royal Society of Chemistry 2025

Electronic Supplementary Information (ESI) for Coherent Collections of Rules Describing Exceptional Materials Identified with a Multi-Objective Optimization of Subgroups

Lucas Foppa*[1] and Matthias Scheffler[1]

[1] *The NOMAD Laboratory at the Fritz Haber Institute of the Max Planck Society, Faradayweg 4-6, D-14195 Berlin, Germany*

(Dated: June 9, 2025)

*foppa@fhi-berlin.mpg.de

Comparison of Approaches for Defining the Pareto Region

In addition to the definition of Pareto region based on the distance to the Pareto front discussed in the main text (hereafter approach A), we have also considered a second approach to define the Pareto region based on the identification of subsequent Pareto fronts, e.g., 2nd, 3rd, or 4th (hereafter approach B). These subsequent N^{th} Pareto fronts are the Pareto fronts that would be identified if the Pareto fronts with indices lower than N would be removed from the pool of solutions. In approach B, the Pareto region is defined as the Pareto front plus one or more of the subsequent Pareto fronts.

Figure S1 compares the Pareto regions obtained by approach A (top) with approach B for the SGs describing perovskites with high bulk modulus obtained using the positive-mean-shift utility function. The figure shows how one can tune the number of SGD solutions in the Pareto region by choosing the threshold distance or the number of subsequent Pareto fronts in approaches A and B, respectively. While there is significant overlap between the Pareto regions identified by the two approaches, there are also some differences. In particular, the SGD solutions with relative size of ca. 0.30 and utility-function values of ca. 0.60, highlighted by the black circle on the top-left panel, are not included in the Pareto region when approach A is used with threshold distances up to 0.03 nor it is captured by approach B considering only the 2nd subsequent Pareto front. However, the mentioned SGD solutions are included in the Pareto region when a sufficiently large threshold distance is chosen in approach A or when more than 3 subsequent Pareto fronts are taken into account in approach B. We note that the domination count, i.e., the number of SGD solutions that dominate a given SGD solution with respect to the two targets to be optimized, could also be used to select the SGD solutions to be included in the Pareto region.

Let us now discuss the advantages and disadvantages of approaches A and B for defining the Pareto region. The definition of Pareto region via a fixed distance to the Pareto front has the disadvantage that it depends on the form of the Pareto front and on the distribution of SGD solutions within the Pareto front. However, approach A has at least one advantage. The larger the distance between two SGD solutions in the size vs. utility-function space, the larger the difference in objective-function value. Thus, by selecting the Pareto region based on a fixed distance to the Pareto front, we ensure that all SGD solutions of the Pareto region have objective-function values within a specific range determined by the chosen threshold distance. Approach B is more robust than approach A with respect to the form of the Pareto front and to the distribution of SGD solutions. However, approach B has the disadvantage of not ensuring that the values of the objective function associated to the solutions in the Pareto region are contained within a specific range. Thus, SGD solutions with objective-function values significantly lower than the optimal one might be included in the Pareto region identified by approach B. The solutions highlighted by the black circle in Figure S1, for instance, have a significantly lower objective-function value compared to the objective-function values of the SGD solutions in the Pareto front.

Analysis of Pareto Region of SG Solutions Based on Similarity Between SG Rules and Hierarchical Clustering

In order to assess the variability of the SG rules within the Pareto region, we define a similarity measure between two SGs using the Jaccard similarity index J:

$$J(SG_i, SG_j) = \frac{|SG_i \cap SG_j|}{|SG_i \cup SG_j|}. \quad (1)$$

$J(SG_i, SG_j)$ corresponds to the number of data points that satisfy the rules associated to two SGs, SG_i and SG_j, divided by the number of data points corresponding to the union of the two SGs. J ranges from 0 to 1, and the higher the value, the more similar the SGs. We use the $J(SG_i, SG_j)$ values to create a *similarity matrix* containing the Jaccard indices for all combinations of SGs in the Pareto region. Obviously, $J(SG_i, SG_j) = J(SG_j, SG_i)$, i.e., this matrix is symmetric.

Then, we group the SGs of the Pareto region according to their similarity by applying agglomerative hierarchical clustering[**?**] to the similarity matrix. In this unsupervised clustering approach, each data point, i.e. each SG, is initially considered a cluster. Then, the two most alike clusters with respect to a linkage criteria are combined (agglomerated), forming a single bigger cluster. This procedure is repeated until all data points are contained in one large cluster. The clustering analysis results in a sorted similarity matrix, where similar SGs are close to each other. Moreover, a tree-like diagram is generated. The bottom of this diagram contains the individual SGs (tree leafs). These leafs are linked by branches that are

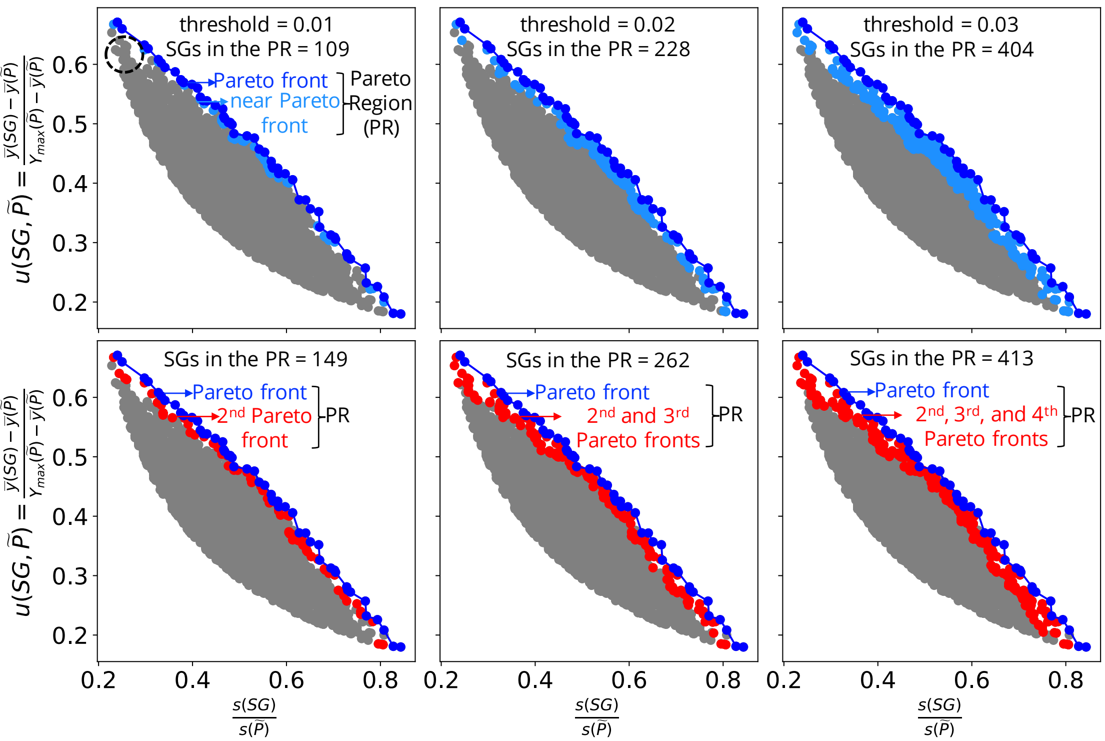

FIG. S1. Comparison of two approaches for defining the Pareto region of SGD solutions for the SGD solutions obtained with the positive-mean-shift utility function and the full feature set containing the 24 features of Table I. (Top): The Pareto region is defined as the Pareto front plus set of solutions within a fixed threshold distance to the Pareto front in the relative SG size-utility function space. The threshold distance can be adjusted to include more or less SGs in the Pareto region. (Bottom): The Pareto region is defined as the Pareto front plus subsequent Pareto fronts.

combined as the height increases. This tree-like structure shows how the individual SGs and clusters were agglomerated at each iteration of the algorithm. It is called dendogram, and it can be truncated at any height to create an arbitrary number of clusters between 1 and the total number of SGs in the Pareto region. Thus, the boundaries between the clusters of SG rules are determined by the sequence of agglomeration steps and by the number of clusters that is chosen. These clusters can then be used to analyze the coherent collection and to choose the SG rules that should be considered for further investigation of physical insights or for the screening of new materials. We note that the number of clusters is not defined beforehand, but one rather chooses the desired number of cluster to consider when truncating the dendogram. In this paper, we used the unweighted pair group method with arithmetic mean as linkage criterion. The Euclidean distance was taken as distance measure. The hierarchical clustering was performed using the seaborn package.[?] We stress that we have chosen hierarchical agglomerative clustering as an example of clustering method. However, other approaches (e.g., k-means clustering) could also be used.

The Jaccard similarity indices for all pairs of SGs of the Pareto region identified with the positive-mean-shift utility function are depicted by the colors in the similarity matrix of Fig. S2(A). In this figure, the SG indices are sorted according to increasing coverage. The regions close to the diagonal are associated to close-to-one $J(SG_i, SG_j)$, indicating that SGs with similar coverages values are similar. Moreover, this plot shows that SGs with low coverage present rather low similarity with the average- and high-coverage SGs. By applying the hierarchical clustering to the similarity matrix (Fig. S2(B)), distinct portions of the Pareto region are more clearly identified. In order to obtain examples of clusters that can identified in this analysis, we truncate the generated dendogram at the height indicated by upper edge of the colored rectangles in Fig. S2(B). We find four different clusters, colored in purple, red, magenta, and orange. These clusters are represented in Fig. 2(B) in the main text with the same colors as in Fig. S2(B).

Variability of the SGD results with respect to dataset size

We evaluate how the Pareto fronts of SGs change with respect to the dataset size by training the SGD with random selections of 75%, 50%, and 25% of the dataset. For each data-set size, five independent random selections were considered. We evaluated the similarity between the Pareto front of SGs obtained with the whole dataset and the Pareto front of SGs obtained with smaller data-set sizes. Fig. S3 display the results for a SGD analysis

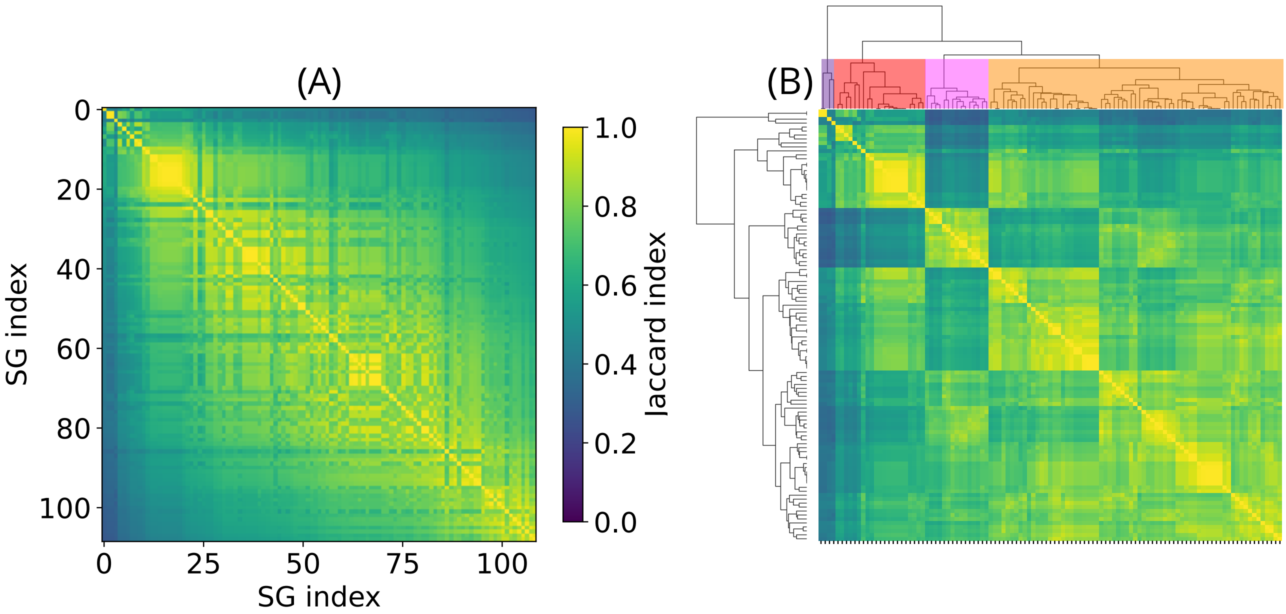

FIG. S2. Similarity analysis of the Pareto front obtained with the positive-mean-shift utility function in Fig. 2 of the main text. (A): Similarity among the 109 SGs of the Pareto region. (B) Hierarchical cluster map obtained by applying agglomerative hierarchical clustering to the similarity matrix shown in (A).

using the positive mean shift utility function and the full feature set. The Jaccard similarity indices decrease with decreasing data-set size. This is particularly the case for SGs presenting low relative sizes. However, the SGs obtained with only 25% of the dataset that present average or high relative size present significant similarity compared to the SGs obtained with the entire dataset. This is indicated by the yellow color at the diagonal of the panels displayed in the third columns of Fig. S3. Thus, for the problem under consideration, SGD would be efficient even with significantly less data.

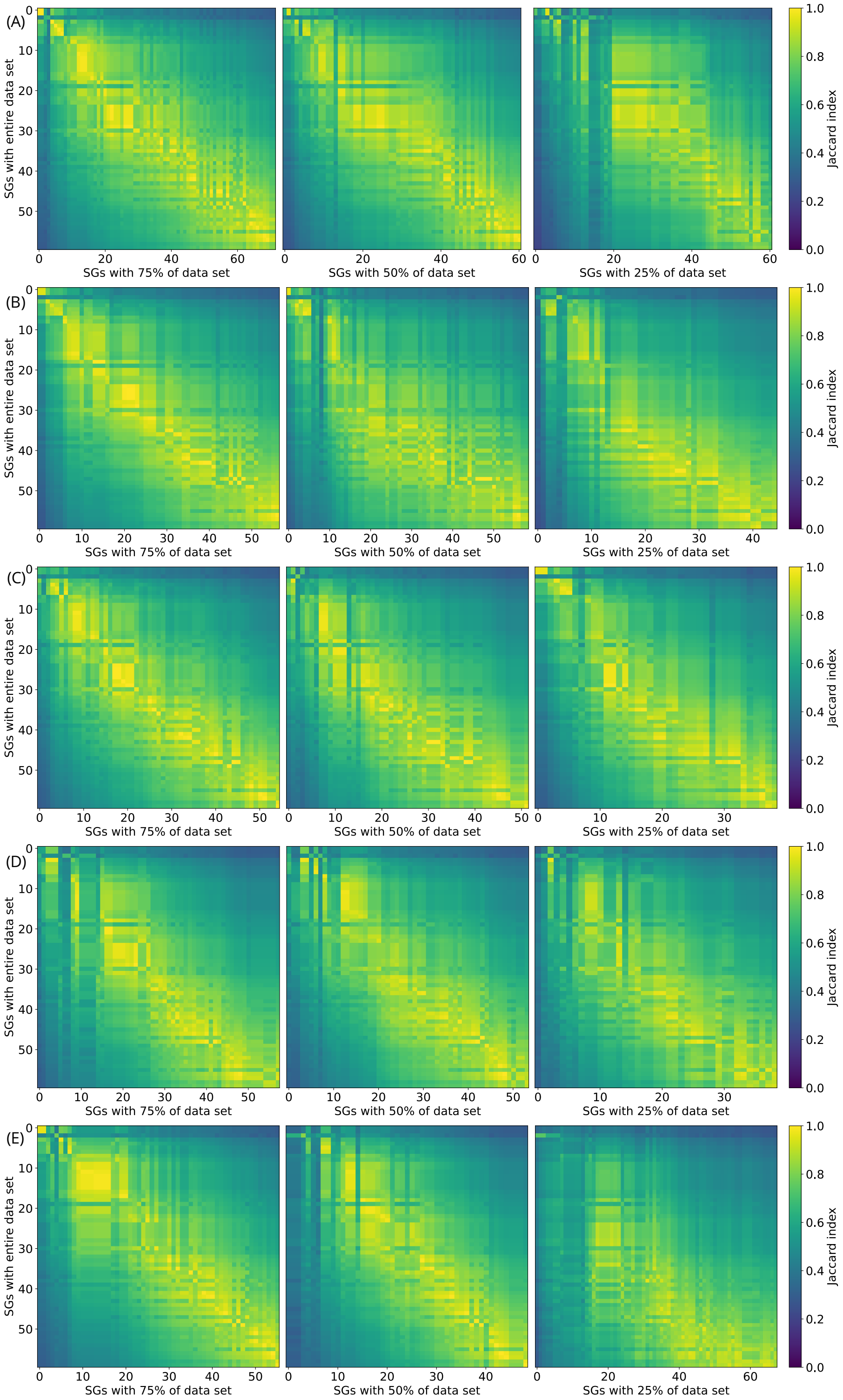

FIG. S3. Jaccard similarity between SGs at the Pareto Front obtained using the entire dataset and using a fraction of the dataset. Results shown for the utility function positive mean shift and the full feature set containing the 24 features. The different panels from (A) to (E) correspond to different random selections of data. In the similarity matrices, the SGs are arranged from low to high relative sizes.
